# Supplementary material for: Actions of FTY720 (Fingolimod), a Sphingosine-1-Phosphate Receptor Modulator, on Delayed-Rectifier K+ Current and Intermediate-Conductance Ca2+-Activated K+ Channel in Jurkat T-Lymphocytes
Source: Molecules. 2020 Oct 2;25(19):4525. doi: 10.3390/molecules25194525 (PMC7582672; doi:10.3390/molecules25194525)
Supplement: Supplementary file 1 [file molecules-25-04525-s001.pdf]

Supplement materials

Data analyses

The concentration-response data for FTY720-induced block of $I_{K(DR)}$ in response to ramp pulse from −90 to +90 mV were fitted with a modified form of the Hill equation. That is,

$$y = 1 - \frac{(1-a)\times[C]^{n_H}}{IC_{50}^{n_H} + [C]^{n_H}},$$

where y is the relative amplitude of $I_{K(DR)}$ measured at the level of +60 mV; [C] is the concentration of FTY720; IC_{50} and n_H are the concentration required for a 50% inhibition and the Hill coefficient, respectively. Maximal inhibition (i.e., 1-a) of $I_{K(DR)}$ in the presence of FTY720 was also estimated.

Blocking and unblocking rate constants (i.e., k_{+1} and k_{-1}) were taken from the inactivation rate constants (1/ $_{inact}$) of $I_{K(DR)}$ in response to rapid depolarizing pulses from -50 to +50 mV with a duration of 1 sec. Blocking and unblocking rate constants were then determined using the following relation:

$$\frac{1}{\tau_{inact}} = k_{+1}\times[B] + k_{-1},$$

where [B] is the FTY720 concentration, and k_{+1} and k_{-1} are respectively derived from the slope and the y-axis intercept at [B]=0 of the linear regression interpolating the reciprocal time constant (1/ $_{inact}$) versus different concentrations (0.01-5 M) of FTY720. Based on k_{+1} and k_{-1}, the value of

dissociation constant (K_D=k_{-1}/k_{+1}) is thereafter derived [12].

The steady-state inactivation curve of $I_{K(DR)}$ with or without addition of FTY720 was plotted as a function of the test potential and fitted to the Boltzmann equation:

$$\frac{I}{I_{max}} = \frac{1}{1+\exp\left(\frac{V-V_{1/2}}{k}\right)},$$

where V is the conditioning potential in mV, $V_{1/2}$ the membrane potential for half-maximal inactivation, and k the slope factor of inactivation curve for $I_{K(DR)}$.
